# Bmp Suppression in Mangrove Killifish Embryos Causes a Split in the Body Axis

**DOI:** 10.1371/journal.pone.0084786

**Published:** 2014-01-30

**Authors:** Sulayman Mourabit, Michael W. Moles, Emma Smith, Ronny van Aerle, Tetsuhiro Kudoh

**Affiliations:** Biosciences, College of Life & Environmental Sciences, University of Exeter, Exeter, United Kingdom; Laboratoire de Biologie du Développement de Villefranche-sur-Mer, France

## Abstract

Bone morphogenetic proteins (Bmp) are major players in the formation of the vertebrate body plan due to their crucial role in patterning of the dorsal-ventral (DV) axis. Despite the highly conserved nature of Bmp signalling in vertebrates, the consequences of changing this pathway can be species-specific. Here, we report that Bmp plays an important role in epiboly, yolk syncytial layer (YSL) movements, and anterior-posterior (AP) axis formation in embryos of the self-fertilizing mangrove killifish, *Kryptolebias marmoratus*. Stage and dose specific exposures of embryos to the Bmp inhibitor dorsomorphin (DM) produced three distinctive morphologies, with the most extreme condition creating the splitbody phenotype, characterised by an extremely short AP axis where the neural tube, somites, and notochord were bilaterally split. In addition, parts of caudal neural tissues were separated from the main body and formed cell islands in the posterior region of the embryo. This splitbody phenotype, which has not been reported in other animals, shows that modification of Bmp may lead to significantly different consequences during development in other vertebrate species.

## Introduction

Patterning of the DV axis is regulated by a differential spatial activation of Bmp signalling during early vertebrate embryogenesis [1], [2]. In zebrafish embryos, from the blastula to the gastrula stage, Bmp promotes the development of ventrally derived tissues such as epidermis, posterior spinal cord, posterior somites, and blood, in the ventral side of the embryo; whereas Bmp inhibited areas in the dorsal side give rise to anterior neural cell fates and the notochord. A graded decrease of Bmp from the ventral side to the dorsal side produces laterally derived structures such as trunk somites and the neural tube [1], [3], [4], [5].

In zebrafish, mutations in genes of this signalling pathway lead to a dorsalized phenotype, i.e. an expansion of dorsal-lateral regions of the gastrula at the expense of ventrally derived structures. Milder phenotypes of mutations in genes involved in Bmp signalling display a reduction in the ventral tail fin as seen in the recessive phenotypes *mini fin/tolloid* and *lost-a-fin/alk8* [6], [7], [8]. With increasing severity, ventrally derived tissues such as blood and tail are not apparent, and dorsal tissues such as the notochord are expanded. For instance, the homozygous *snailhouse*/*bmp7* phenotype is characterised by a shortened anterior-posterior axis, which twists around itself posteriorly like a coiled snail shell [6], [9], [10]. In the most severe homozygous phenotypes, such as *swirl/bmp2b* and *somitabun/smad5*, anterior somites expand dramatically constricting the yolk and causing it to burst [6], [10], [11].

Although the Bmp signalling pathway is highly conserved in vertebrates, modified Bmp activity can have different consequences depending on the morphological and genetic characteristics of the species. For example, whereas zebrafish chordino mutants, which have a null mutation in the gene coding for the Bmp-antagonist Chordin, have a significantly smaller brain [12], Chordin knock-out mice do not show such clear reduction in brain size, possibly due to a differential redundancy of another Bmp-antagonist, Noggin [13].

To examine the evolutionary conserved and divergent role of the Bmp signalling pathway and to further investigate the stage specific role of Bmp in vertebrate embryos, we examined Bmp inhibition in the self-fertilizing mangrove killifish *K. marmoratus*. This species is a unique hermaphroditic vertebrate that reproduces primarily by internal self-fertilization, essentially creating clonal lines with homozygous progeny. Mangrove killifish are also androdioecious, meaning that populations are composed of males and hermaphrodites [14]. This reproductive system involves three distinct phenotypes: primary males, hermaphrodites, and secondary males. Primary males (i.e. developed with unisexual male gonads) are rare in most of the wild populations [15]. There are currently 21 distinct clonal lines available for research, of which 11 have been demonstrated to be truly isogenic [16]. The self-fertilizing ability of mangrove killifish makes this species very interesting for mutant screening as zygotic mutant phenotypes appear in F2 embryos, one generation earlier than other animals such as zebrafish and medaka [17]. To establish the species as a novel model for developmental biology, we have recently analysed developmental stages and also developed basic embryological techniques for gene expression, cell labelling, imaging, and chemical treatment [18], [19]. However, the role of key signalling pathways during early development in *K. marmoratus* has not yet been investigated.

Here, we show that a reduction of Bmp activity in *K. marmoratus* achieved by addition of the specific inhibitor DM resulted in significantly different morphological defects compared with the phenotypes previously reported in zebrafish [6], [20]. It is noted that mangrove killifish and zebrafish shared a common ancestor over 250 million years ago, making this is a broad evolutionary comparison.

## Results

### Stage Specific Inhibition of Bmp Induces a Split Body Axis in *K. marmoratus* Embryos

To investigate the role of the Bmp signalling pathway during early embryonic development in *K. marmoratus*, we treated the embryos with the Bmp inhibitor DM [20]. It has been reported that the *snailhouse* phenotype can be produced in zebrafish with early DM exposures (10 µM) [20]. However the authors have not reported severer phenotypes such as the *swirl/bmp2b* mutant phenotype, which presents a more elongated embryo at the bud to early somitogenesis stage and also embryonic lethality during somitogenesis, suggesting that the dose used was not strong enough to suppress all Bmp signalling. Therefore, in the present study, we have used a higher dose of DM (100 µM) to examine severer loss of function of Bmp. Using this dose, zebrafish embryos show the *swirl* mutant phenotype (Cruz et al., in preparation). In addition, to investigate the stage specific role of Bmp, *K. marmoratus* embryos were treated with DM at various stages ranging from cleavage to gastrula.

**Figure 1:**
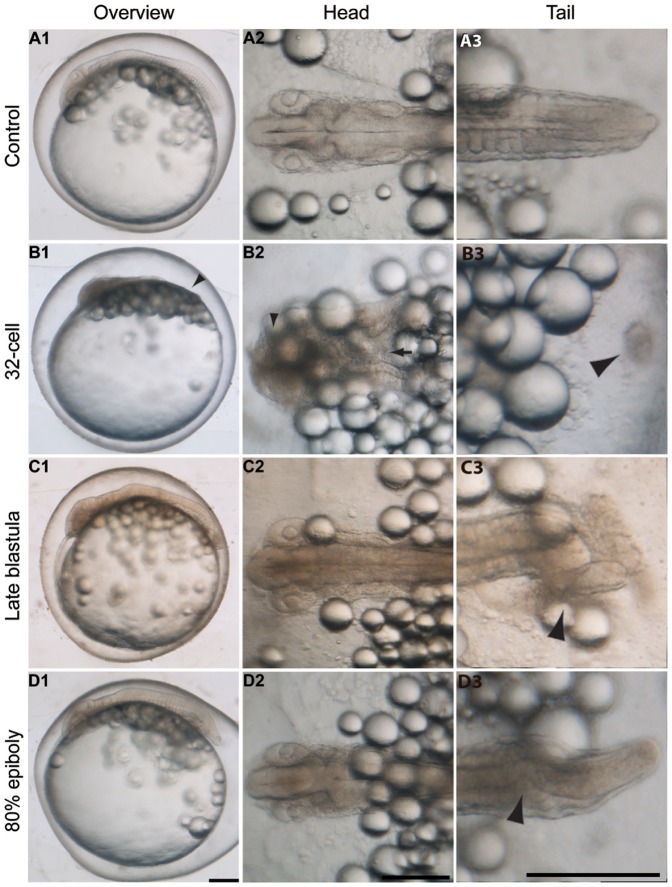
Stage specific inhibition of Bmp in *K. marmoratus*. Embryos were exposed to 100 µM dorsomorphin at the 32-cell (**B**), late blastula (**C**) and 80% epiboly (**D**) stages of development. Photographs of the embryos were taken 3 days post-fertilization. **A1–3**: Control (n = 20/20). **B1–3**: splitbody (phenotype variation details in Figure 3), this phenotype is characterised by absence of a distinct tail region (**B1** arrowhead), morphologically undifferentiated head region (**B2** arrowhead) and split body axis (**B2** arrow), and cell islands in the posterior region (**B3** arrowhead). **C1–3**: Curled tail (n = 8/10), this phenotype resembles *snailhouse* seen in zebrafish and is characterised by its curled tail (**C3** arrowhead). **D1–3**: Bent tail (n = 12/12, this phenotype primarily displayed a bent tail (**D3** arrowhead). Overview images are lateral views and head/tail images are dorsal views of the embryos. Scale bars: 250 µm.

A phenotype resembling *snailhouse* was observed in *K. marmoratus* by exposing embryos to DM from the late blastula stage (Figure 1C) (see [18] for *K. marmoratus* developmental stages), displaying a shortened and curled tail (Figure 1 C3 arrowhead) by 3 days post-fertilization (dpf). Embryos treated with the same concentration but starting from late epiboly (Figure 1D) produced a milder phenotype characterised by its bent tail (Figure 1 D3 arrowhead). However, DM exposures from the 32-cell stage (Figure 1B) produced a distinctive phenotype, hereby referred to as splitbody, characterised by its short body (Figure 1 B1), morphologically undifferentiated head region (Figure 1 B2 arrowhead), split body axis (Figure 1 B2 arrow) and cell clumps (hereby referred to as cell islands) in the posterior region of the embryo (Figure 1 B3 arrowhead). This splitbody phenotype has not been reported in other model species including zebrafish, *Xenopus*, chick and mouse.

### Bmp is Essential for Normal Epiboly Progression in *K. marmoratus*

**Figure 2:**
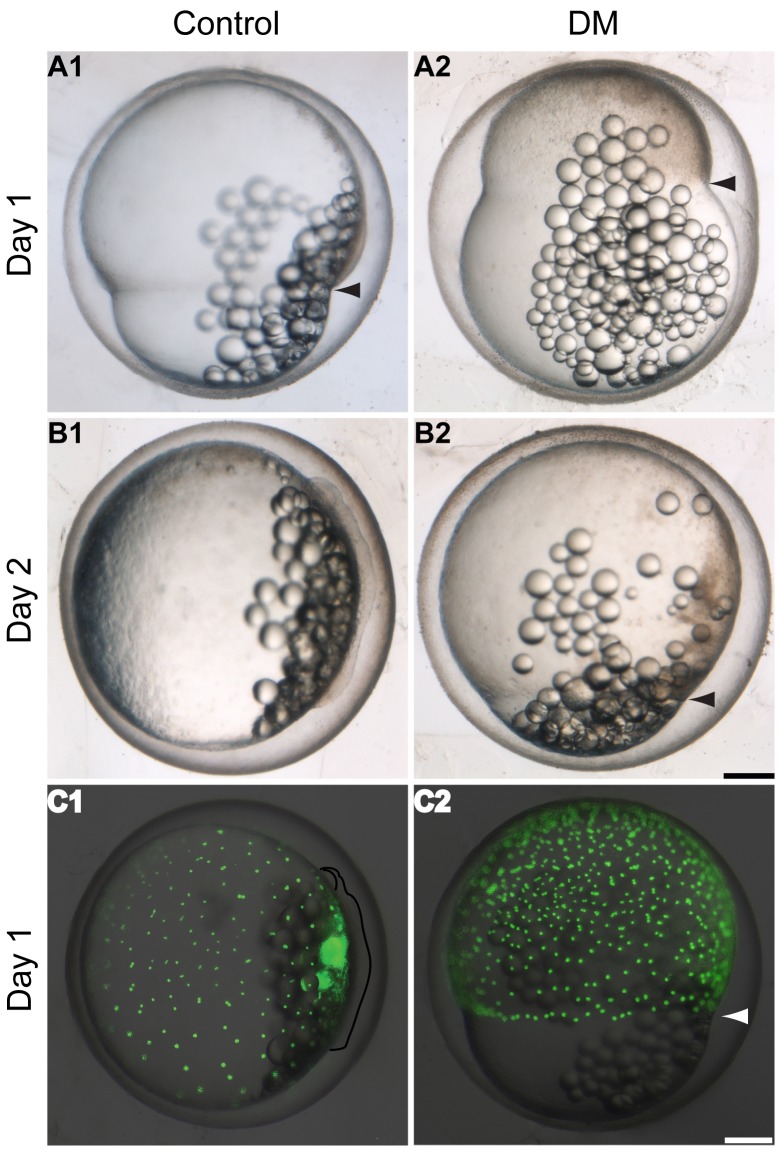
Bmp inhibition delays epiboly progression in *K. marmoratus*. Epiboly coverage was recorded at day 1 (**A**) and day 2 (**B**) post-fertilization (dpf) in embryos exposed to 100 µM dorsomorphin (DM) at the 32-cell stage. Progression of the yolk syncytial layer (YSL) during gastrulation was assessed via staining of yolk syncytial nuclei (YSN) using Sytox Green. The green fluorescent YSN were observed 1 dpf (**C**). **A1, 2**: As control embryos reach *c.* 70% epiboly (**A1** arrowhead, n = 10/10), DM treated embryos are delayed with epiboly covering *c.* 30% of the yolk (**A2** arrowhead, n = 10/10). **B1, 2**: Controls reach the otic vesicle formation stage (**B1,** n = 10/10) whilst exposed embryos are lagging behind around 90% epiboly (**B2** arrowhead, n = 10/10). **C1**, **2**: Shortly after epiboly closure, control embryos enter the eye formation stage (**C1,** n = 10/10) (embryo and the eye are outlined) and YSN are spread all over the yolk. On the other hand DM exposed embryos are still mid-epiboly and fluorescent YSN are observed near the blastoderm margin (**C2** arrowhead, n = 10/10), demonstrating that YSN are also delayed by inhibition of Bmp signalling. All images are lateral views of the embryos. Scale bars: 250 µm.

To determine the cause of a divided AP axis in the splitbody phenotype, we first examined differences in early development between wild type and embryos exposed to 100 µM DM at the 32-cell stage (Figure 2). During gastrulation, Bmp inhibition was shown to clearly delay epiboly progression. At 1 dpf, when control embryos reached *c.* 70% epiboly (Figure 2 A1), DM treated embryos were delayed with epiboly covering *c.* 30% of the yolk (Figure 2 A2). Similarly at 2 dpf, control embryos entered the otic vesicle formation stage (Figure 2 B1), whilst exposed embryos lagged behind with epiboly covering *c.* 90% of the yolk (Figure 2 B2). Such a significant delay in epiboly, resulting from Bmp signalling inhibition, has not been reported in zebrafish embryos [6].

**Figure 3:**
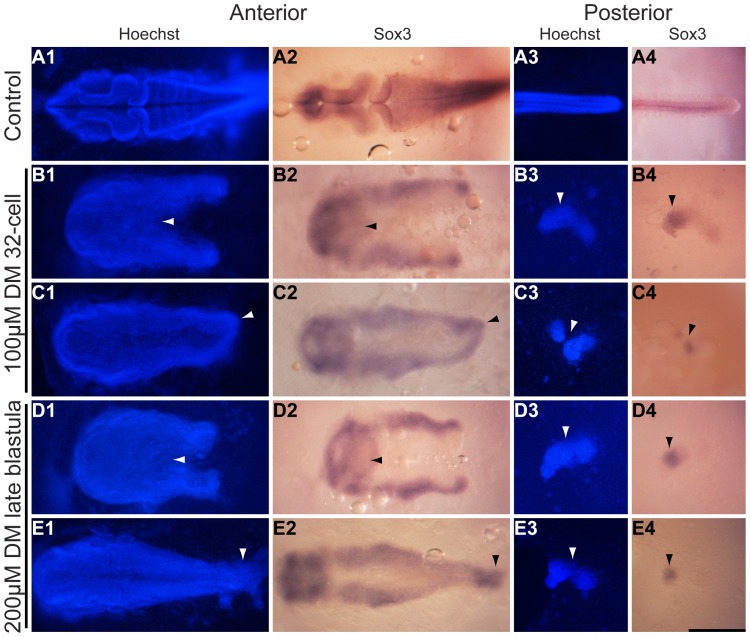
The neural tube is separated in embryos of the splitbody phenotype. *K. marmoratus* embryos were exposed to 100 µM dorsomorphin (DM) at the 32-cell stage (**B, C**), and 200 µM DM at the late blastula stage (**D**, **E**) of development. These embryos were then fixed 3 days post-fertilization and used for *in situ* hybridization using a *sox3* probe (stains all neural tissue) (**A–E2, A–E4**) and Hoechst staining (a blue fluorescent DNA stain) (**A–E1, A–E3**) in order to examine body contour and split neural tube (**A–E1**, **2**), and the nature of the posterior isolated cell lumps or cell islands (**A–E3**, **4**). **A1–4**: Control embryo (n = 20/20). **B, D**: Splitbody individual with an opened end of the body axis and neural tube split (**B1, 2** arrowheads n = 19/20, and **D1, 2** arrowheads n = 12/20). Splitbody individuals with a closed end, as both strands of the body axis and neural tube join in their most posterior region (**C1, 2** arrowheads n = 1/20, and **E1, 2** arrowheads n = 8/20). All DM embryos presented here generated cell islands (**B-E3** arrowheads) with distinct *sox3* positive staining (**B–E4** arrowheads). All images are dorsal views of the embryos. Scale bar: 250 µm.

Prior to gastrulation, the embryo is composed of 4 layers, the enveloping layer, deep cells, the yolk syncytial layer (YSL) and the yolk [21]. It is known that in zebrafish, during late gastrulation, delays in movements of the deep cells do not equate to delays in the YSL [22]. Furthermore, research in *Fundulus* has shown that the movements of the YSL are independent from the blastoderm, as the YSL continues its epibolic migration if the blastoderm is removed [23]. Thus, in order to determine if inhibition of Bmp signalling also delayed YSL movements, yolk syncytial nuclei (YSN) were stained by sytox green injection at the late blastula stage. Embryos were then exposed to 200 µM DM, a concentration capable of mimicking the splitbody phenotype (see the next section and Figure 3). Embryos were observed the next day, and whilst controls reached the eye formation stage with YSN spread throughout the yolk (Figure 2 C1), both the YSN and the blastoderm of DM exposed embryos were delayed at mid-epiboly (Figure 2 C2). This data suggests that the YSL is also affected by the inhibition of Bmp, as YSN were moving relative to the blastoderm margin and displayed the same level of delay.

### Laterally Derived Structures and the Notochord are Divided in Splitbody

**Figure 4:**
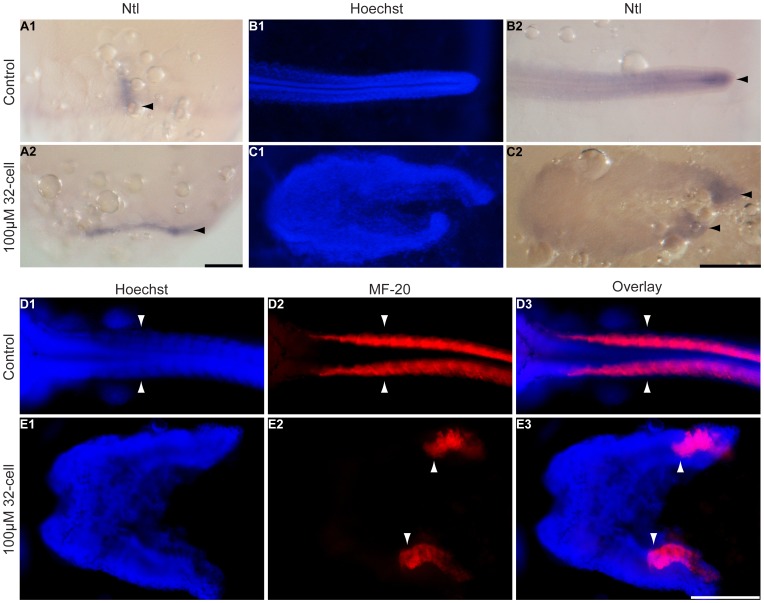
Somites and the notochord are divided in the splitbody phenotype. *K. marmoratus* embryos were exposed to 100 µM dorsomorphin at the 32-cell stage and fixed 1 and 4 days post-fertilization in order to stain the notochord by *in situ* hybridization using a medaka *ntl* probe (**A, B**, **C**), and somites using the myosin antibody MF-20 (**D**, **E**). In control embryos at late gastrula, *ntl* stained axial mesoderm in the dorsal axis (**A1** arrowhead, n = 10/10), whereas in DM treated embryos these cells appeared to have stayed in the lateral domains (**A2** arrowhead, n = 10). At day 4, *ntl* stained the notochord in the tip of the tail for control embryos (**B2** arrowhead, n = 10/10), whereas splitbody embryos had the tips of both body axes stained with *ntl* (**C2** arrowheads, n = 10/10). For control embryos, somites are formed as pairs arranged either side of the neural axis (**D1–3** arrowheads, n = 10/10). In the splitbody phenotype, somites were unpaired and separated in the two body axes (**E1** Hoechst staining showing the body split; **E2, 3** arrowheads, somites are present in both axes, n = 10/10). Photographs were taken at late gastrula for **A**, and 4 days post-fertilization for **B**–**E**. Images in **A** are lateral views and for **B**–**E** dorsal views of the embryos. Scale bars: 250 µm.

The severe delay of epiboly movements observed in splitbody embryos suggests that laterally derived structures are unable to merge at the end of epiboly, leading to the formation of two body axes. To confirm this hypothesis, we examined the spatial arrangement of the neural tube and somites in splitbody (Figure 3&4). These two tissues may be derived from lateral gastrula domains, as reported in zebrafish [3]. We used Hoechst staining for the body contour, *sox3* and *ntl in situ* hybridization for the neural tube and the notochord respectively, and MF-20 immunofluorescence staining for the somites.

Hoechst and *sox3* staining confirmed a split in the body axis and the neural tube for all embryos exposed to both 100 µM DM at the 32-cell stage and 200 µM DM at the late blastula stage. Two different phenotypes were observed, with some individuals showing an opened end of the two neural tube strands (Figure 3 B1, 2 & D1, 2 arrowheads), whilst others had a closed end with both strands joining in their most posterior section (Figure 3 C1, 2 & E1, 2 arrowheads). Significantly more individuals with the former phenotype were observed in the 100 µM DM treated embryos (19/20 individuals) compared to the 200 µM DM treated embryos (12/20; P = 0.02). Furthermore, the cell islands seen in splitbody were observed by Hoechst staining (Figure 3 B-E3 arrowheads), and displayed *sox3* positive staining suggesting that the island is partly composed of neural plate cells (Figure 3 B-E4 arrowheads).

At the late gastrula stage, the axial mesoderm of normal embryos stained by *ntl* was observed in the dorsal axis (Figure 4 A1 arrowhead), whereas in DM treated embryos these cells appeared to stay separated in the lateral domains (Figure 4 A2 arrowhead). At day 4 in control embryos, the tip of the notochord was stained by *ntl* (Figure 4 B2 arrowhead). On the other hand, splitbody embryos possessed two *ntl* stained tips (Figure 4 C2 arrowheads), suggesting that the axial mesoderm cells separated in the lateral domains of the gastrula embryo were split into the two body axes. This separation of lateral structures was further demonstrated by the staining of somite muscles in embryos 4 dpf using the myosin antibody MF-20. If epiboly occurs correctly, somites form pairs either side of the neural tube of the developing embryos (Figure 4 D1–3 arrowheads). In the splitbody phenotype, somites were unpaired and appeared divided in the two strands of the embryonic body axis (Figure 4 E1 Hoechst staining showing the clear split of the body axis; E2, 3 arrowheads, somites are present in both strands of the divided body). In the cell islands, *ntl* and MF-20 did not show any staining suggesting these cells do not contain notochord, muscle, and undifferentiated mesoderm.

### DM Dose Dependence of the Splitbody Phenotype

**Figure 5:**
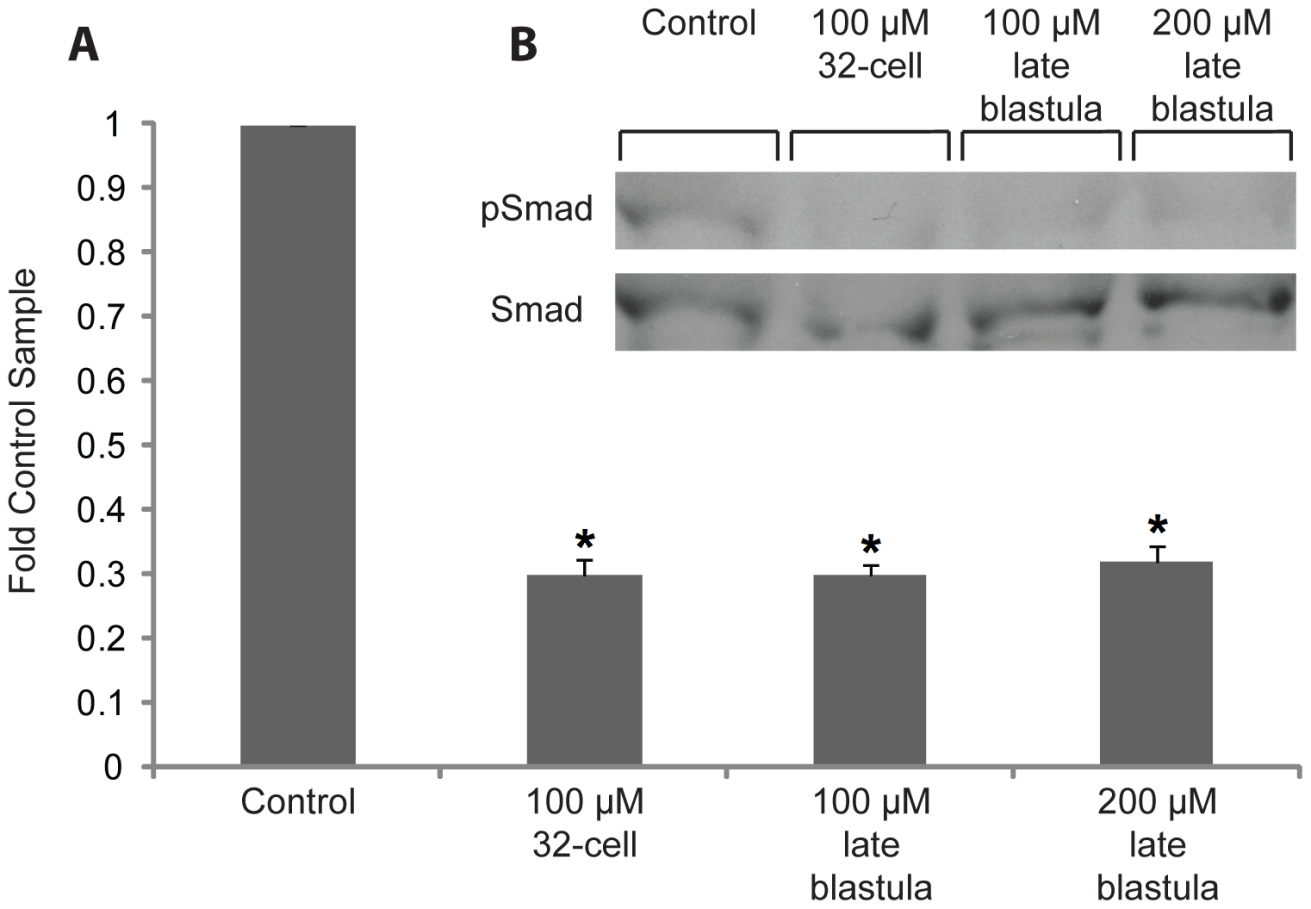
Dorsomorphin inhibits phosphorylation of Smad1/5. Bmp signalling activity was quantified by measuring phosphorylation of Smad 1/5 at the late gastrula stage. Embryos were exposed to 100 µM from 32-cell as well as 100 and 200 µM DM from late blastula. These were then frozen at the late gastrula stage and used for Western Blotting. **A:** Quantification of densitometry results obtained from 3 independent experiments (Mean ± SE), normalised to total Smad and indicated as fold increase over the resting control condition. All 3 treatments were significantly different from the control as indicated by asterisks (P<0.001), but no significant differences were observed between the treatments. **B:** Representative Western Blot of 3 independent experiments showing the levels of total Smad1/5/8 and phospho-Smad1/5 at late gastrula from the dose and stage specific treatments. These data demonstrate that all 3 treatments equally suppress phospho-Smad1/5 by late gastrula.

Given that embryos treated to 100 µM DM from 32-cell and 200 µM DM from late blastula displayed a similar splitbody phenotype, we hypothesised that the 100 µM dose took longer to fully suppress Bmp signalling, but as embryos were treated earlier in development Bmp was fully suppressed by the mid-blastula transition and produced splitbody. To explore this hypothesis, we examined the level of Bmp signalling activity by measuring phosphorylation of Smad1/5 with Western blotting. Embryos were exposed to 100 µM from 32-cell as well as 100 and 200 µM DM from late blastula. These were then frozen at the late gastrula stage and used for Western Blotting. Quantification of densitometry results was obtained from 3 independent experiments, normalised to total Smad and indicated as fold increase over the resting control condition (Figure 5A, Mean ± SE). The representative Western Blot of the 3 independent experiments (Figure 5B) shows the levels of total Smad1/5/8 and phospho-Smad1/5 at late gastrula from the dose and stage specific treatments. These data demonstrate that all three treatments equally suppress phospho-Smad1/5 by late gastrula. These results confirmed that DM effectively suppressed Bmp signalling during gastrulation, but also suggested that zygotic Bmp is key for normal epiboly movements, as the 100 µM treatment only produced splitbody if applied earlier in development.

## Discussion

Here, we report a defect in the merging of laterally/ventrally derived structures at the end of epiboly and a division of the notochord, as a result of Bmp signalling inhibition. Both neural tube and somites were unable to completely merge at the end of gastrulation due to delayed epiboly and YSL movements, thus producing the splitbody phenotype.

The YSL plays an important role in epiboly movements of the blastoderm. Studies in *Fundulus* have shown that although epiboly can take place after removal of the blastoderm, the latter is unable to perform this morphogenetic movement without the YSL [23], [24]. Here, we demonstrate that the delayed blastoderm was accompanied by a delay in movements of the YSN. It is unclear whether the delay in movements of the YSL is triggered by the setback of the blastoderm, but the relative independence of the YSL from the blastoderm discussed previously suggests that Bmp plays a crucial role in movements of the YSL during gastrulation.

It is known from zebrafish research that the Bmp gradient of the zebrafish gastrula regulates convergent extension (CE), as these morphogenetic movements are absent ventrally whilst lateral tissues display increased CE until the dorsal side where convergence weakens and extension stays strong [25]. This pattern corroborates with and is driven by the ventrally-high, laterally-gradient, and dorsally-low distribution of Bmp signalling during gastrulation [25]. It is thought that this Bmp gradient in the zebrafish gastrula regulates CE movements by applying a reverse gradient of cell adhesion in which Bmp signalling negatively affects the Cadherin-dependent adhesion of lamellipodia from mesodermal cells to adjacent cells. This creates a spatial gradient of cell-cell adhesion that directs the lamellipodia-driven cell migration of lateral regions towards the dorsal side, as cell movement via lamellipodia is increasingly disabled as Bmp signalling increases [26]. Here, splitbody *K. marmoratus* embryos experienced low to absent levels of Bmp throughout the gastrula thus disturbing CE movements. In the absence of strong convergence and a gradient of cell-cell adhesion, lumps of cells could be isolated from the main body axis. In addition, the weakened extension movements explain the extremely shortened body axis of the splitbody phenotype.

The variation observed in splitbody, where some individuals exhibited an opened double body axis and others a closed one, resulted from the stage and dose specific exposure of *K. marmoratus* embryos to DM, with the more mild treatments producing the *snailhouse* equivalent. Splitbody was only produced when embryos were treated early in development (100 µM at 32-cell stage or 200 µM at late blastula) to ensure a delay of epiboly and thus a split in the body axis. These results suggested that zygotic Bmp is key for normal epiboly movements, as the 100 µM treatment only produced splitbody if applied earlier in development, thus giving DM the time to fully suppress Bmp by the onset of epiboly. On the other hand, it may also be possible that maternal Bmp acts as an activator to the zygotic signalling pathway; as such the lower dose earlier in development could affect the late blastula to gastrula stages. Recent studies have shown the temporal importance of Bmp signalling in patterning DV tissues along the AP axis [2]. Our data demonstrate that Bmp signalling is also crucial for the correct timing of epiboly closure and thus the formation of a single anterior-posterior body axis in the mangrove killifish. Such results have not been reported in zebrafish, suggesting that the suppression of Bmp may have different consequences during development in other fish species.

The mangrove killifish produces eggs roughly two times bigger than zebrafish. Consequently the ratio between the diameter of the blastoderm margin and the yolk is 1∶1.4 for killifish Stage 10 embryos and 1∶1 in zebrafish embryos at an equivalent stage (shield stage) [18], [21]. As the blastoderm is under extreme tension to move over the yolk during epiboly, the larger yolk of *K. marmoratus* embryos may increase the stretch required for the sheet of deep cells to reach the mid-gastrula point, which may enhance the epiboly defect less obvious in zebrafish. Furthermore, the differential genetic background of this species may result in varying levels of severity of the patterning defect caused by Bmp suppression. Both a genome project and a mutant screen are currently ongoing for the mangrove killifish [17], [27], and will help further uncover the molecular mechanisms and phenotypic variation of the loss of function of Bmp signalling between different species.

It has been demonstrated that DM may inhibit other receptors of the Alk family [28], therefore it might be possible that part of the splitbody phenotype is due to inhibition of other pathways. However, if DM inhibits Alk receptors transducing Nodal/Activin, the mesoderm marker *ntl* would have been suppressed, which was not the case. The Bmp specificity of the chemically induced phenotype is also supported by the phenotype of DM treated zebrafish embryos, as these are identical to *bmp2b* and *bmp7* mutants ([10], [29]; Cruz et al., in preparation). Therefore in the context of early development it is likely that the main target of inhibition causing the splitbody phenotype is the Bmp pathway.

In summary, by using a novel model animal we were able to find a very different morphological phenotype when blocking Bmp signalling. In zebrafish, most embryos affected by a severe suppression of Bmp die by late somitogenesis, whereas mangrove killifish embryos at an equivalent stage all survived and displayed a very unique splitbody phenotype, enabling analysis for both early and late phenotypes resulting from a severe reduction of Bmp signalling. These data provide new insights for the conserved and species-specific roles of Bmp at the blastula, gastrula, and somitogenesis stages, demonstrating that the mangrove killifish is a very useful model for studying the roles of Bmp and other key signalling pathways in early development.

## Experimental Procedures

### Experimental Animals

*K. marmoratus* of the Hon9 clonal lineage were obtained from an existing stock at the University of Exeter (UK). Hermaphroditic individuals were kept individually in 1.5 L plastic containers (25°C, 14 psu (practical salinity unit), 12∶12 h light:dark photoperiod) and were fed daily *ad libitum* on *Artemia* nauplii. Brackish water was made using demineralised water and marine salts (Tropic Marin, Germany). Eggs were collected from aquaria filter pads placed in the containers (Pondmaster filter foams), and provided a substrate for oviposition. Both control and DM treated embryos were reared under the same conditions as adult individuals. Embryonic stages were determined using the staging series in [18]. All Protocol used were permitted by the UK Home Office guidance to Animals Scientific Procedures Act (Project License No. PPL 30/2360).

### Experimental Protocols

#### Imaging

Micrographs were taken using a Nikon Digital Sight DS-U2 camera mounted on a Nikon SMZ1500 microscope and an Olympus XC10 camera mounted on an Olympus SZX16 microscope. Imaging of live and fixed *K. marmoratus* embryos was performed using the Agarose bed and methyl cellulose techniques respectively, described in detail by [18], [19].

#### Dorsomorphin exposures

Stock solutions for dorsomorphin (DM) (6-(4-(2-(1-Piperidinyl)ethoxy]phenyl]-3-(4-pyridinyl)-pyrazolo(1,5-*a*]pyrimidine dihydrochloride, Tocris 3093) were prepared as 10 mM dissolved in demethyl sulfoxide (DMSO) and diluted in 14 psu brackish water to final concentrations. Embryos at the 32-cell, late blastula and 80% epiboly stages were exposed to different concentrations of DM (100 µM and 200 µM).

#### Whole-mount *in situ* hybridization

*K. marmoratus sox3* was cloned by PCR using the following primers: forward GAGTGTGTGAGTGATCACTGA, and reverse TCTGAGAGTGGGACGTGATGG. Primer design was based on *sox3* sequence information that was obtained from a *de novo K. marmoratus* transcriptome assembly (Illumina RNA-seq sequencing) conducted in our laboratory. *K. marmoratus Sox3* cDNA was sequenced and deposited to Genbank (KF887913).

The PCR product was inserted into the pGEM-T Easy vector (Promega). *Escherichia coli* colonies containing this plasmid were cultured and the plasmid DNA was then purified using a QIAprep Spin Midiprep (QIAGEN). The plasmids were digested with *PstI*, and Digoxigenin-labelled RNA probe was synthesised by T7 RNA polymerase (Roche). The medaka *ntl* plasmids [30] were digested with *SalI*, and the Digoxigenin-labelled RNA probe was synthesised by T3 RNA polymerase (Roche).

Live *K. marmoratus* embryos with chorions were placed in 1.5 ml Eppendorf tubes (5 embryos/tube). After removing brackish water with a pipette, 1 ml of 4% paraformaldehyde (PFA) (14 psu brackish water, 20 mM HEPES buffer, pH adjusted to 7) was added for fixation and kept at room temperature for 4 days. Following fixation, these embryos were washed with 1 ml phosphate buffer saline (10 minutes) then manually dechorionated and dehydrated in 1 ml 100% methanol at −20°C for one hour (they can be stored at this step for several weeks). These embryos were then used for whole-mount *in situ* hybridization, performed according to the method described by [31], with modifications. A full protocol is available in the supplemental information section (Supporting Information S1).

Differences between the phenotypes observed after DM treatment (Figure 3) were determined using Fischer Exact Tests (Systat Software, San Jose, CA). Differences between groups were considered to be significant when P<0.05.

#### Hoechst and immunofluorescent staining

For MF-20 antibody staining (Hybridoma Bank), fixed embryos stored in methanol (see above for conditions), were rehydrated in PBSTx (PBS+0.5% Tritonx, Sigma) and further permeabilised using Proteinase K (PK). Control and DM treated embryos at 3 dpf were treated to 10 µg/ml PK for 5 minutes, and day 4 embryos for 10 minutes. These embryos were then washed in PBSTx to stop the digestion and re-fixed with 4% PFA for an hour at room temperature. Embryos were put in blocking solution for 3 hours at room temperature (1% skimmed milk and 1% DMSO in PBSTx), and then incubated in primary antibody overnight (1∶20 monoclonal mouse antibody MF-20 in blocking solution). The next day, the primary antibody was thoroughly washed off (four 30 minute washes in PBSTx), and incubated in Alexa Fluor 546 goat anti-mouse IgG secondary antibody overnight (Invitrogen A11003). Finally, the secondary antibody was thoroughly washed off.

For Hoechst staining, *sox3* or MF-20 stained embryos were incubated in a Hoechst solution for 30 minutes (0.5 µg/ml in PBSTx). The solution was then thoroughly washed off and embryos were ready for imaging.

#### Microinjection

Microinjection of sytox green (Invitrogen S7020) into the yolk syncytial layer (YSL) was performed following the procedure described by [18], [19]. Sytox green (0.5 mM) was injected in the YSL at the late blastula stage and fluorescent yolk syncytial nuclei were photographed at 1 dpf in control and DM treated embryos.

#### Western blotting

Embryos at the late blastula stage were lysed in cold 2× lysis buffer (4% SDS, 20% glycerol, 125 mM Tris-HCl pH 6.8, 50 µg/ml BPB, 10% β-Mercaptoethanol) at 5 embryos/400 µl lysis buffer. Lysates were clarified by centrifugation (14.5 Krpm for 5 minutes) and the supernatants were heated at 70°C for 10 minutes then analysed by SDS-PAGE. Western Blots for Smad1/5/8 (1∶200; Santa Cruz Biotechnology sc-6031-R) and Phospho-Smad1/5 (1∶1000: New England Biolabs 9516S) were performed according to the manufacturer’s instructions (blocking solution, for pre-blocking and dilution of all the antibodies, was composed of 2% bovine serum albumin in Tris buffer saline (20 mM Tris-Hcl pH 7.5 and 150 mM NaCl). Differences in phospho-Smad1/5 densitometry results between experimental groups were determined using One Way ANOVA, followed by pair-wise comparisons between DM-treated embryos and the controls using the Holm-Sidak method (Systat Software, San Jose, CA). Differences between groups were considered to be significant when P<0.05.

## Supporting Information

Supporting Information S1 Document detailing mangrove killifish whole-mount in situ hybridization”.
(DOCX)
