# Supplementary material for: Bmp Suppression in Mangrove Killifish Embryos Causes a Split in the Body Axis
Source: PLoS One. 2014 Jan 30;9(1):e84786. doi: 10.1371/journal.pone.0084786 (PMC3907431; doi:10.1371/journal.pone.0084786)
Supplement: Supporting Information S1 — Document detailing mangrove killifish whole-mount in situ hybridization”. (DOCX) [file pone.0084786.s001.docx]

**Supplemental Information**

**Mangrove killifish whole-mount *in situ* hybridization**

Embryos were fixed in 1 ml paraformaldehyde (4% PFA (Sigma), artificial sea salts at 14 psu (Tropic Marin), 20 mM HEPES (Sigma), pH 7) at the desired stage in 1.5 ml Eppendorf tubes for 4 days at room temperature (5 embryos/tube). After fixation, PFA was removed and the embryos were washed with 1 ml of phosphate buffer saline (PBS) for 10 minutes. During this wash the embryos were dechorionated with fine tweezers. Dechorionated embryos were then transferred to methanol by a series of gradual changes (25, 50, 75% in PBTw (1x PBS, 0.1% Tween20) for 1 minute each, then left in 100% methanol at -20 °C for one hour (this step can be used to store embryos for several weeks).

Methanol was then gradually washed (75, 50, 25% MeOH in PBTw for 1 minute each) and the embryos were then left in 1 ml of PBTw for 10 minutes.

(Optional *stage*: further permeabilisation of the embryos can be achieved by exposing them to proteinase K (Sigma) (10 μg/ml, in PBTw) at room temperature for 5 minutes (3 days post-fertilization embryos or older, timings may change). Embryos were then washed with PBTw for 5 minutes (twice), fixed in 4% PFA for 1 hour, then washed with PBTw 5 minutes (twice).]

Embryos were then pre-hybridized in 0.5 ml Hyb buffer at 65 °C for one hour (Hyb buffer: 50% formamide, 5x saline sodium citrate (SSC), 5 mM EDTA, 0.1% Tween20, 0.1% CHAPS, 50 μg/ml heparin, 1 mg/ml torula RNA). Hyb buffer was then replaced with 0.2 – 0.5 ml of probe/Hyb buffer and left at 65 °C overnight (the diluted probe in Hyb buffer was preheated at 80 - 90 °C for 10 minutes and immediately chilled on ice for 5 minutes before it was used).

The probes were recovered the next day (they can be used a few times), and embryos were washed with 1 ml of washing solution 1 (50% formamide, 2x SSC, 0.1% Tween20) at 65 °C for 30 minutes. They were then washed with 1 ml washing solution 2 (2x SSC, 0.1% Tween20) at 65 °C for 30 minutes. Finally the embryos were washed with 1 ml washing solution 3 (0.2x SSC, 0.1% Tween20) at 65 °C for 30 minutes (this last was performed twice).

Embryos were then washed with 1 ml PBTw at room temperature for 10 minutes. The samples were then placed in 0.5 ml blocking solution for one hour. Blocking solution is prepared with 1 g blocking reagent (Roche) dissolved in 47.5 ml maleic acid buffer (MAB), and 2.5 ml of lamb serum was then added when the solution had cooled down. MAB: 0.1 M maleic acid, 150 mM NaCl, pH to 7.5.

Embryos were then placed in 0.2 - 0.5 ml anti-DIG solution (diluted 1:100 in blocking solution) for 2 hours. The anti-DIG solution was made by mixing 10 μl Anti-DIG antibody conjugated with alkaline phosphatase (Roche) with 500 μl of blocking solution and 20 fixed zebrafish embryos (bud, somitogenesis or 24 hour stage; these must be PFA fixed, methanol treated then PBS rehydrated). This solution was left to shake gently for 4 hours at 4 °C before use.

The embryos were then washed in 1 ml PBTw for 30 minutes (four times). The last wash can be done overnight at 4 °C. For the last wash, the embryos were placed in a 24 well-plate using a large-mouth glass pipette (heated to polish the rim). The PBTw was then removed and 1 ml alkaline phosphatase (AP) buffer was added for 10 minutes at room temperature on a shaker (AP buffer: 0.1 M Tris pH 9.5, 0.1 M NaCl, 50 mM MgCl_2_, 0.1% Tween20). AP buffer was then replaced with 0.5 ml BM purple (Roche). The plate was placed in a opaque box to shut off the light and left for 30 minutes to overnight on a shaker, periodically checking the embryos until the staining comes up (overnight staining should be done at 4 °C). Once the embryos were displaying sufficient staining, BM purple was removed and the embryos washed with 1 ml PBTw for 10 minutes, then fixed in 4% PFA.
